# 7-Methyl-5-[(4-methyl­benzene)­sulfon­yl]-2*H*,5*H*-[1,3]dioxolo[4,5-*f*]indole: crystal structure and Hirshfeld analysis

**DOI:** 10.1107/S2056989018000889

**Published:** 2018-01-19

**Authors:** Akbar Ali, Julio Zukerman-Schpector, Márcio Weber Paixão, Mukesh M. Jotani, Edward R. T. Tiekink

**Affiliations:** a Departamento de Química, Universidade Federal de São Carlos, 13565-905 São Carlos, SP, Brazil; b Laboratório de Cristalografia, Esterodinâmica e Modelagem Molecular, Departamento de Química, Universidade Federal de São Carlos, 13565-905 São Carlos, SP, Brazil; c Department of Physics, Bhavan’s Sheth R. A. College of Science, Ahmedabad, Gujarat 380001, India; d Centre for Crystalline Materials, School of Science and Technology, Sunway University, 47500 Bandar Sunway, Selangor Darul Ehsan, Malaysia

**Keywords:** crystal structure, indole, 1,3-dioxole, Hirshfeld surface analysis

## Abstract

In the title indole derivative, C_17_H_15_NO_4_S, the fused dioxolo-indole system is essentially planar [r.m.s. deviation of the 12 fitted atoms = 0.0249 Å] and is effectively perpendicular to the appended 4-tolyl ring, forming a dihedral angle of 89.95 (6)°. Overall, the mol­ecule has the shape of the letter L. In the crystal, supra­molecular layers in the *ab* plane are formed *via* weak 4-tolyl-C—H⋯π(C_6_-ring of indole) and S—O⋯π(1,3-dioxole) contacts. The aforementioned inter­actions along with inter­atomic H⋯H and H⋯O contacts are all shown to make significant contributions to the calculated Hirshfeld surfaces.

## Abstract 2

The mol­ecule in the title compound has the shape of the letter L. In the crystal, weak 4-tolyl-C—H⋯π(C_6_-ring of indole) and S—O⋯π(1,3-dioxole) contacts link the mol­ecules into a supra­molecular layer in the *ab* plane.

## Chemical context   

Nitro­gen-based heterocycles comprise a class of compounds with significant biological importance that are crucial in organic synthesis (Trofimov *et al.*, 2004 ▸). In particular, indole and oxindole derivatives continue to receive significant attention in both contexts as these residues are found in both natural products as well as in synthetic drugs (Dalpozzo, 2015 ▸). Not surprisingly, considerable effort is continually being made to develop new and efficient methods for their synthesis. Recently, the development of a useful method for the synthesis of indoles and oxindoles was described (da Silva *et al.*, 2015 ▸). The protocol was based on a combination of tris­(tri­methyl­sil­yl)silane, as the hydride source, and visible light to promote intra­molecular reductive cyclization of suitable precursors. Among the compounds synthesized in this study was the title compound (I), which features an indole residue N-bound to a (4-methyl­benzene)­sulfonyl, *i.e*. tosyl, residue and fused to a 1,3-dioxole ring at the benzene ring. Herein, the crystal and mol­ecular structures of (I) are described along with an analysis of the calculated Hirshfeld surfaces.
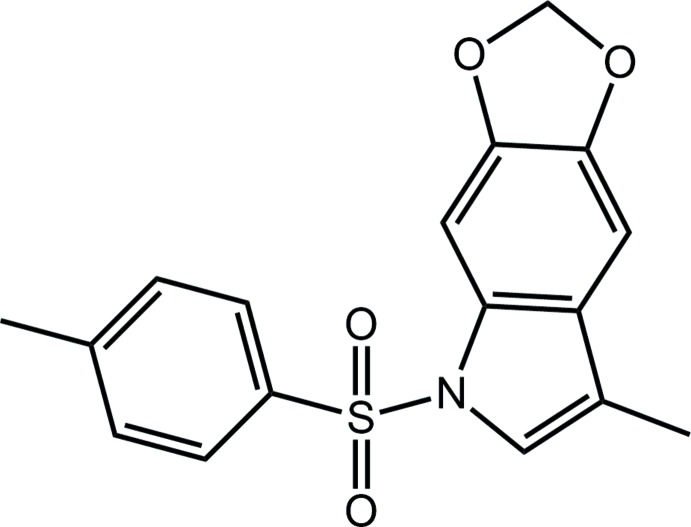


## Structural commentary   

**Figure 1:**
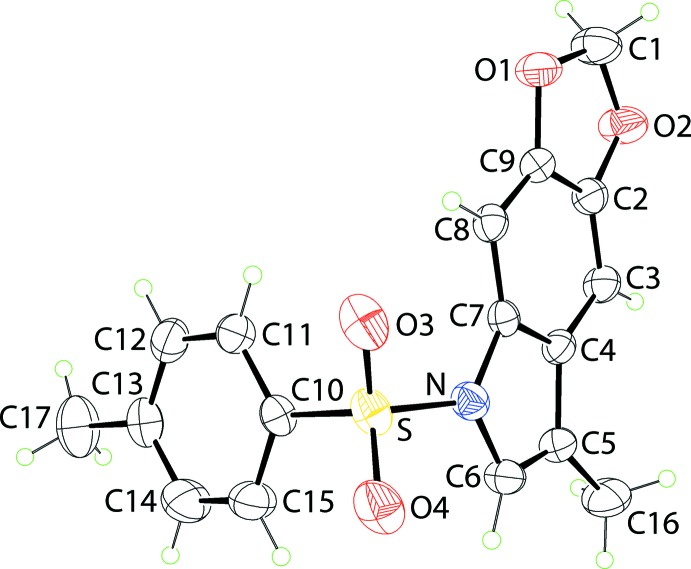
The mol­ecular structure of (I), showing the atom-labelling scheme and displacement ellipsoids at the 35% probability level.

The mol­ecular structure of (I), Fig. 1 ▸, comprises two essentially planar residues, *viz*. 4-tolyl and the fused dioxolo-indole system, hinged at the SO_2_ group. The r.m.s. deviation of the five non-hydrogen atoms comprising the 1,3-dioxole ring is 0.0158 Å with the maximum deviations above and below this plane being 0.022 (14) and 0.021 (14) Å for the C1 and O2 atoms, respectively. This planarity extends over the entire dioxolo-indole residue, which exhibits an r.m.s. deviation of 0.0249 Å for the 12 constituent atoms with maximum deviations of 0.058 (2) and 0.0284 (14) Å for the C1 and C8 atoms, respectively. The dihedral angle between the residues linked at the S atom is 89.95 (6)°, *i.e.* indicating a perpendicular relationship consistent with the shape of the letter L. The CNO_2_ atoms about the S atom define a tetra­hedron with widest angle being subtended by the doubly bonded O3 and O4 atoms, *i.e*. O3—S—O4, is 120.32 (10)°.

## Supra­molecular features   

**Figure 2:**
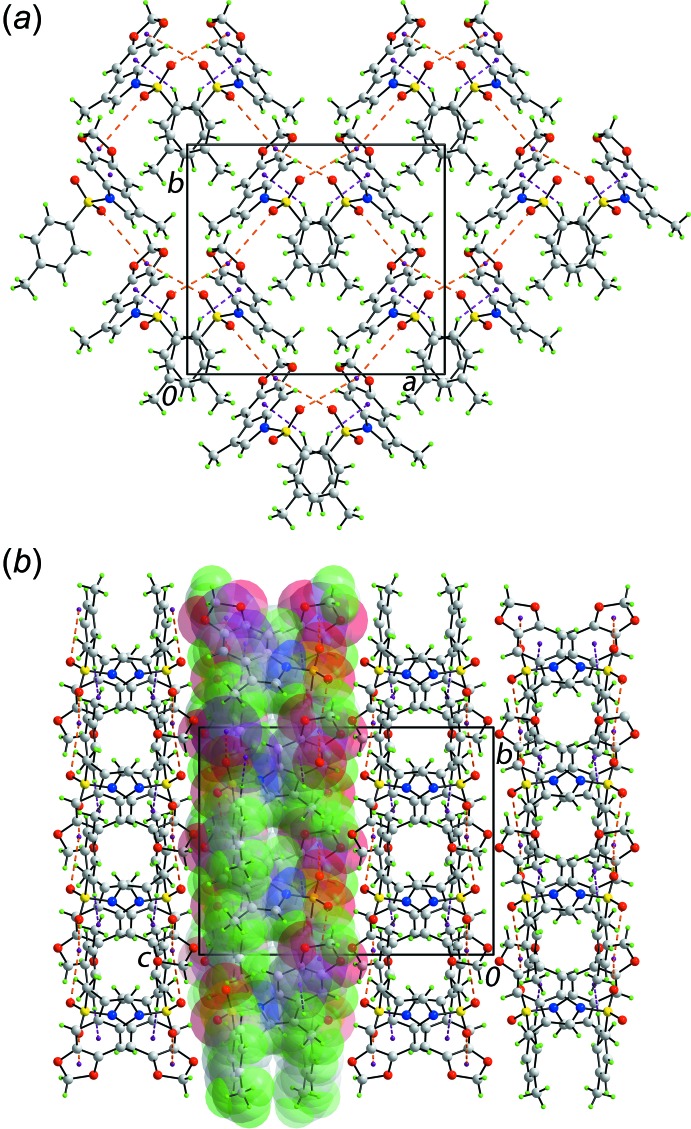
Mol­ecular packing in (I): (*a*) view of the supra­molecular layer in the *ab* plane and (*b*) the unit-cell contents shown in projection down the *a* axis; one layer is highlighted in space-filling mode. The C—H⋯π and S—O⋯π contacts are shown as purple and orange dashed lines, respectively.

The mol­ecular packing of (I) features a number of weak inter­molecular contacts with the weaker ones discussed below in *Analysis of the Hirshfeld surface* (§4). Three specific points of contact between mol­ecules are highlighted here, *i.e.* within the standard distance criteria in PLATON (Spek, 2009). These are: a 4-tolyl-C11—H11⋯π(C2–C4,C7–C9) contact and a pair of S—O⋯π(1,3-dioxole) contacts, Table 1 ▸, implying the 1,3-dioxole ring serves as a bridge between two symmetry-related mol­ecules. These inter­actions cooperate to form a supra­molecular layer in the *ab* plane as shown in Fig. 2 ▸ *a*. Layers stack along the *c* axis with no directional inter­actions between them, Fig. 2 ▸ *b*.

## Hirshfeld surface analysis   

**Figure 3:**
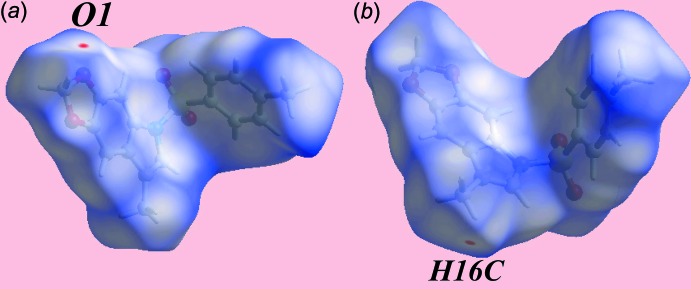
Two views of the Hirshfeld surface mapped over *d*_norm_ for (I) in the range −0.039 to +1.643 au.

**Figure 4:**
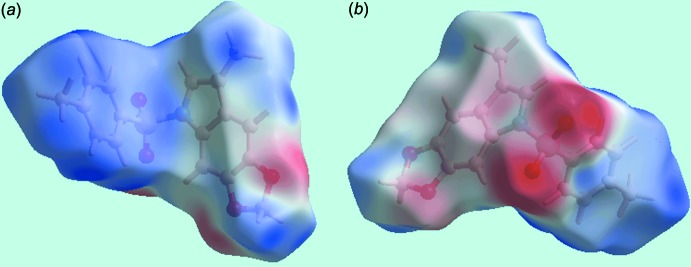
Two views of the Hirshfeld surface mapped over the electrostatic potential for (I) in the range ±0.075 au.

**Figure 5:**
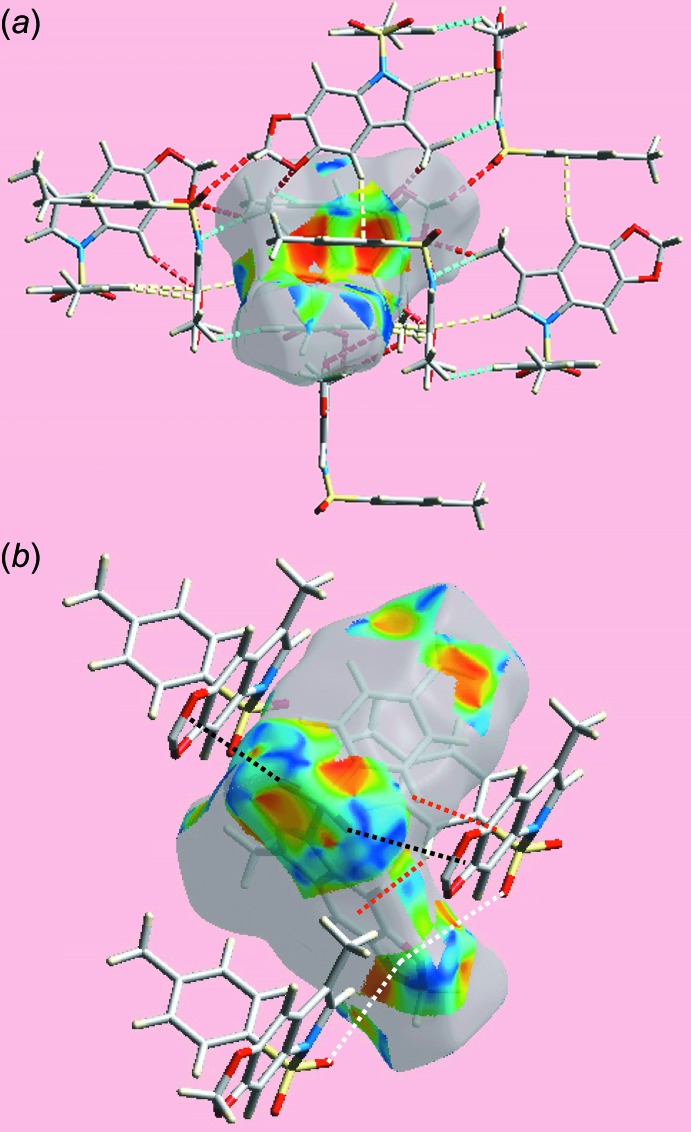
Two views of the Hirshfeld surface about reference mol­ecule of (I) mapped with the shape-index property highlighting (*a*) H⋯H, O⋯H/H⋯O and C⋯H/H⋯C contacts by sky-blue, red and yellow dashed lines, respectively, and (*b*) C—H⋯π/π⋯H—C contacts by red dashed, S—O⋯π and its reciprocal, *i.e*. π⋯O—S, contacts by black and white dashed lines, respectively.

The Hirshfeld surfaces calculated for (I) were performed in accord with a recent report on a related organic mol­ecule (Zukerman-Schpector *et al.*, 2017 ▸) and provide an explanation of the influence of short inter­atomic contacts upon the mol­ecular packing in the absence of conventional hydrogen bonding. The donor and acceptor of the relatively weak inter­atomic C—H⋯O inter­action, summarized in Table 2 ▸, are viewed as diminutive-red spots near methyl-H16*C* and dioxole-O1 on the Hirshfeld surfaces mapped over *d*_norm_ in Fig. 3 ▸; this contact occurs in the inter­layer region along the *c* axis. On the Hirshfeld surfaces mapped over the electrostatic potential, Fig. 4 ▸, the blue and red regions are assigned to positive and negative potentials, respectively. Views of the Hirshfeld surfaces about a reference mol­ecule mapped within the shape-index property highlighting short inter­atomic H⋯H, O⋯H/H⋯O, C⋯H/H⋯C, C—H⋯π/π⋯H—C and S—O⋯π/π⋯O—S contacts, Tables 1 ▸ and 2 ▸, are highlighted in Fig. 5 ▸.

**Figure 6:**
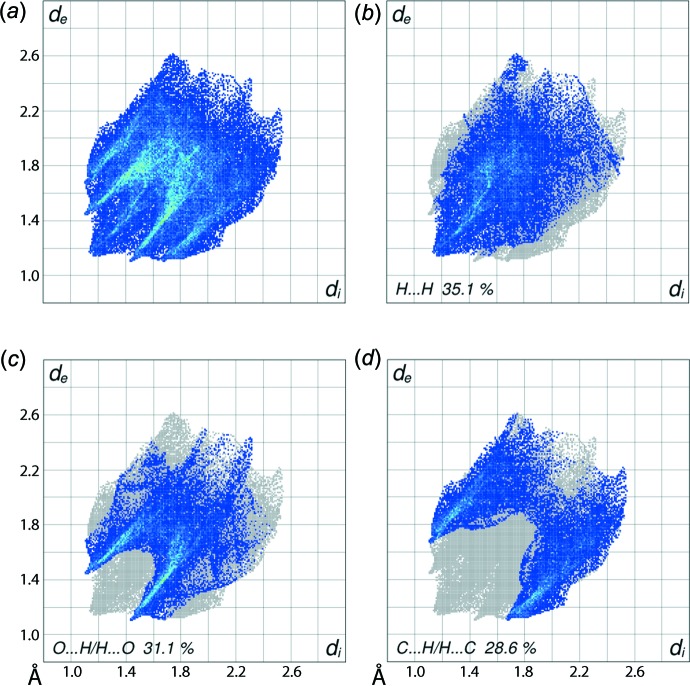
(*a*) The full two-dimensional fingerprint plot and fingerprint plots delineated into (*b*) H⋯H, (*c*) O⋯H/H⋯O and (*d*) C⋯H/H⋯C contacts for (I).

The overall two-dimensional fingerprint plot for (I) is shown in Fig. 6 ▸ *a* and those delineated into H⋯H, O⋯H/H⋯O and C⋯H/H⋯C contacts (McKinnon *et al.*, 2007 ▸) are shown in Fig.6*b*–*d*. All plots illustrate the influence of the short inter­atomic contacts in the crystal. The percentage contributions from the different inter­atomic contacts to the Hirshfeld surfaces are summarized in Table 3 ▸ and indicate that H⋯H, O⋯H/H⋯O and C⋯H/H⋯C contacts all make quite significant contributions as a result of the short inter­atomic contacts listed in Tables 1 ▸ and 2 ▸. These inter­atomic contacts are viewed as the distribution of points with the pair of tips at *d*_e_ + *d*_i_ ∼ 2.2, 2.6 and 2.8 Å in their respective delineated fingerprint plots, Fig. 6 ▸ *b*–*d*. The inter­molecular C—H⋯π contact involving the tolyl-C11 atom and the fused (C2–C4,C7–C9) ring is viewed as the pair of characteristic wings in the fingerprint plot delineated into C⋯H/H⋯C contact, Fig. 6 ▸ *d*. The presence of a pair of inter­molecular S—O⋯π contacts in the crystal is also indicated by small but significant contributions from C⋯O/O⋯C and O⋯O contacts to the Hirshfeld surface, Table 3 ▸. The contribution from C⋯C and N⋯H/H⋯N contacts do not have a great influence on the mol­ecular packing as their inter­atomic separations are greater than sum of their respective van der Waals radii.

## Database survey   

The N-bound tosyl and methyl group substitution pattern, flanking the central hydrogen atom, in the five-membered ring of the indole residue, as in (I), has one precedent in the literature, namely, a derivative with a benzo­yloxy substituent in the indole-benzene ring, *i.e*. 5-benz­yloxy-3-methyl-1-tosyl-1*H*-indole (Pozza Silveira *et al.*, 2013 ▸). On the other hand, there are several more examples where a 1,3-dioxole ring has been fused to the indole-benzene ring. A closely related species to (I) has two such fused ring systems linked *via* a C(=O)—C(=O) bridge and with each nitro­gen bound to a benzyl group, *i.e*. 1,2-bis­[5-benzyl-5*H*-(1,3)dioxolo(4,5-*f*)indole-6-yl]ethane (Lindsay *et al.*, 2007 ▸); the mol­ecule has twofold symmetry. To a first approximation, the conformations of the ring systems in the cited literature structures matches that observed in (I).

## Synthesis and crystallization   

The compound was prepared and characterized as described in the literature (da Silva *et al.*, 2015 ▸). Irregular, colourless, crystals of (I) for the X-ray study were obtained by slow evaporation from its ethanol solution.

## Refinement details   

Crystal data, data collection and structure refinement details are summarized in Table 4 ▸. The carbon-bound H atoms were placed in calculated positions (C—H = 0.93–0.97 Å) and were included in the refinement in the riding-model approximation, with *U*_iso_(H) set to 1.2–1.5*U*_eq_(C).

## Supplementary Material

Crystal structure: contains datablock(s) I, global. DOI: 10.1107/S2056989018000889/hb7727sup1.cif

Structure factors: contains datablock(s) I. DOI: 10.1107/S2056989018000889/hb7727Isup2.hkl

CCDC reference: 1816872

Additional supporting information:  crystallographic information; 3D view; checkCIF report

## Figures and Tables

**Table 1:** Hydrogen-bond geometry (Å, °) *Cg*1 and *Cg*2 are the centroids of the (O1,O2,C1,C2,C9) and (C2–C4,C7–C9) rings, respectively.

*D*—H⋯*A*	*D*—H	H⋯*A*	*D*⋯*A*	*D*—H⋯*A*
C11—H11⋯*Cg*2^i^	0.93	2.88	3.662 (2)	142
S—O3⋯*Cg*1^i^	1.42 (1)	3.77 (1)	4.9921 (12)	144 (1)
S—O4⋯*Cg*1^ii^	1.43 (1)	3.86 (1)	4.9243 (12)	132 (1)

Symmetry codes: (i) 
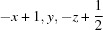
; (ii) 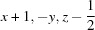.

**Table 2:** Summary of short inter­atomic contacts (Å) in (I)

Contact	Distance	Symmetry operation
H8⋯H16*C*	2.23	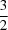 − *x*, − 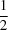 + *y*, 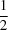 − *z*
H14⋯H16*A*	2.33	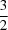 − *x*, 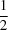 + *y*, 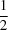 − *z*
O1⋯H8	2.61	1 − *x*, *y*, 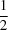 − *z*
O1⋯H16*C*	2.54	− 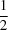 + *x*, − 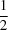 + *y*, *z*
O2⋯H16*B*	2.62	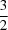 − *x*, 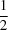 − *y*, 1 − *z*
O3⋯H1*B*	2.63	*x*, − *y*, − 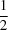 + *z*
C4⋯H12	2.84	1 − *x*, *y*, 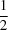 − *z*
C9⋯H6	2.78	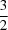 − *x*, 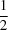 + *y*, 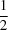 − *z*
C9⋯H11	2.86	1 − *x*, *y/*, 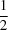 − *z*

**Table 3:** Percentage contributions of inter­atomic contacts to the Hirshfeld surface for (I)

Contact	Percentage contribution
H⋯H	35.1
O⋯H/H⋯O	31.1
C⋯H/H⋯C	28.6
N⋯H/H⋯N	2.4
C⋯C	1.7
C⋯O/O⋯C	0.7
O⋯O	0.4

**Table 4:** Experimental details

Crystal data	
Chemical formula	C_17_H_15_NO_4_S
*M*_r_	329.36
Crystal system, space group	Monoclinic, *C*2/*c*
Temperature (K)	290
*a*, *b*, *c* (Å)	15.2673 (13), 12.5337 (9), 17.6096 (15)
β (°)	112.628 (3)
*V* (Å^3^)	3110.3 (4)
*Z*	8
Radiation type	Mo *K*α
μ (mm^−1^)	0.23
Crystal size (mm)	0.35 × 0.28 × 0.17

Data collection	
Diffractometer	Bruker APEXII CCD
Absorption correction	Multi-scan (*SADABS*; Sheldrick, 1996 ▸)
*T*_min_, *T*_max_	0.709, 0.745
No. of measured, independent and observed [*I* > 2σ(*I*)] reflections	27617, 3198, 2784
*R*_int_	0.028
(sin θ/λ)_max_ (Å^−1^)	0.625

Refinement	
*R*[*F* ^2^ > 2σ(*F* ^2^)], *wR*(*F* ^2^), *S*	0.041, 0.125, 1.07
No. of reflections	3198
No. of parameters	210
H-atom treatment	H-atom parameters constrained
Δρ_max_, Δρ_min_ (e Å^−3^)	0.26, −0.27

Computer programs: *APEX2* and *SAINT* (Bruker, 2009 ▸), *SIR2014* (Burla *et al.*, 2015 ▸), *SHELXL2014/6* (Sheldrick, 2015 ▸), *ORTEP-3 for Windows* (Farrugia, 2012 ▸), *DIAMOND* (Brandenburg, 2006 ▸), *MarvinSketch* (ChemAxon, 2010 ▸) and *publCIF* (Westrip, 2010 ▸).

## supplementary crystallographic information

### Crystal data

**Table:**

C_17_H_15_NO_4_S	*F*(000) = 1376
*M**_r_* = 329.36	*D*_x_ = 1.407 Mg m^−^^3^
Monoclinic, *C*2/*c*	Mo *K*α radiation, λ = 0.71073 Å
*a* = 15.2673 (13) Å	Cell parameters from 9928 reflections
*b* = 12.5337 (9) Å	θ = 2.2–26.4°
*c* = 17.6096 (15) Å	µ = 0.23 mm^−^^1^
β = 112.628 (3)°	*T* = 290 K
*V* = 3110.3 (4) Å^3^	Irregular, colourless
*Z* = 8	0.35 × 0.28 × 0.17 mm

### Data collection

**Table:**

Bruker APEXII CCD diffractometer	2784 reflections with *I* > 2σ(*I*)
φ and ω scans	*R*_int_ = 0.028
Absorption correction: multi-scan (SADABS; Sheldrick, 1996)	θ_max_ = 26.4°, θ_min_ = 2.2°
*T*_min_ = 0.709, *T*_max_ = 0.745	*h* = −19→19
27617 measured reflections	*k* = −15→15
3198 independent reflections	*l* = −22→21

### Refinement

**Table:**

Refinement on *F*^2^	0 restraints
Least-squares matrix: full	Hydrogen site location: inferred from neighbouring sites
*R*[*F*^2^ > 2σ(*F*^2^)] = 0.041	H-atom parameters constrained
*wR*(*F*^2^) = 0.125	*w* = 1/[σ^2^(*F*_o_^2^) + (0.0609*P*)^2^ + 2.5262*P*] where *P* = (*F*_o_^2^ + 2*F*_c_^2^)/3
*S* = 1.07	(Δ/σ)_max_ = 0.001
3198 reflections	Δρ_max_ = 0.26 e Å^−^^3^
210 parameters	Δρ_min_ = −0.27 e Å^−^^3^

### Special details

**Table:**

Geometry. All esds (except the esd in the dihedral angle between two l.s. planes) are estimated using the full covariance matrix. The cell esds are taken into account individually in the estimation of esds in distances, angles and torsion angles; correlations between esds in cell parameters are only used when they are defined by crystal symmetry. An approximate (isotropic) treatment of cell esds is used for estimating esds involving l.s. planes.

### Fractional atomic coordinates and isotropic or equivalent isotropic displacement parameters (Å^2^)

**Table:**

	*x*	*y*	*z*	*U*_iso_*/*U*_eq_	
C1	0.63451 (17)	−0.0477 (2)	0.44548 (14)	0.0694 (7)	
H1B	0.5872	−0.0417	0.4695	0.083*	
H1A	0.6615	−0.1188	0.4565	0.083*	
C2	0.71138 (13)	0.08717 (16)	0.41601 (11)	0.0460 (4)	
C3	0.77317 (13)	0.16699 (16)	0.41880 (11)	0.0471 (4)	
H3	0.8199	0.1899	0.4679	0.057*	
C4	0.76150 (11)	0.21227 (14)	0.34255 (11)	0.0398 (4)	
C5	0.81363 (13)	0.29544 (15)	0.32141 (12)	0.0474 (4)	
C6	0.77434 (14)	0.30697 (15)	0.23940 (13)	0.0511 (5)	
H6	0.7939	0.3563	0.2097	0.061*	
C7	0.68969 (11)	0.17599 (13)	0.26958 (10)	0.0363 (4)	
C8	0.62689 (12)	0.09373 (14)	0.26740 (11)	0.0399 (4)	
H8	0.5796	0.0698	0.2190	0.048*	
C9	0.64139 (12)	0.05175 (14)	0.34288 (11)	0.0414 (4)	
C10	0.54356 (13)	0.35424 (15)	0.12094 (11)	0.0450 (4)	
C11	0.46506 (13)	0.33127 (17)	0.13887 (12)	0.0513 (5)	
H11	0.4494	0.2610	0.1450	0.062*	
C12	0.41023 (14)	0.41411 (19)	0.14760 (13)	0.0585 (5)	
H12	0.3576	0.3989	0.1600	0.070*	
C13	0.43162 (16)	0.51894 (19)	0.13835 (14)	0.0603 (5)	
C14	0.51076 (19)	0.53963 (19)	0.12083 (19)	0.0789 (8)	
H14	0.5265	0.6100	0.1149	0.095*	
C15	0.56675 (18)	0.45869 (18)	0.11193 (18)	0.0695 (7)	
H15	0.6197	0.4741	0.1000	0.083*	
C16	0.89722 (17)	0.3540 (2)	0.38141 (17)	0.0742 (7)	
H16A	0.9423	0.3037	0.4161	0.111*	
H16B	0.8764	0.4002	0.4146	0.111*	
H16C	0.9265	0.3958	0.3519	0.111*	
C17	0.3709 (2)	0.6088 (2)	0.1472 (2)	0.0907 (9)	
H17A	0.4101	0.6595	0.1865	0.136*	
H17B	0.3241	0.5809	0.1658	0.136*	
H17C	0.3400	0.6432	0.0949	0.136*	
N	0.69912 (11)	0.23364 (12)	0.20445 (10)	0.0447 (4)	
O1	0.59147 (10)	−0.03034 (12)	0.35900 (9)	0.0605 (4)	
O2	0.70666 (11)	0.02948 (14)	0.48058 (9)	0.0708 (5)	
O3	0.56284 (12)	0.15436 (12)	0.09072 (9)	0.0636 (4)	
O4	0.66542 (13)	0.28779 (14)	0.06197 (10)	0.0718 (5)	
S	0.61611 (4)	0.25106 (4)	0.11113 (3)	0.04973 (18)	

### Atomic displacement parameters (Å^2^)

**Table:**

	*U*^11^	*U*^22^	*U*^33^	*U*^12^	*U*^13^	*U*^23^
C1	0.0734 (14)	0.0767 (15)	0.0543 (13)	−0.0216 (12)	0.0204 (11)	0.0117 (11)
C2	0.0420 (9)	0.0550 (11)	0.0375 (9)	−0.0013 (8)	0.0116 (7)	0.0063 (8)
C3	0.0406 (9)	0.0561 (11)	0.0371 (9)	−0.0072 (8)	0.0067 (7)	−0.0005 (8)
C4	0.0341 (8)	0.0407 (9)	0.0423 (9)	−0.0017 (7)	0.0121 (7)	−0.0019 (7)
C5	0.0416 (9)	0.0446 (10)	0.0557 (11)	−0.0046 (8)	0.0184 (8)	0.0015 (8)
C6	0.0528 (11)	0.0455 (10)	0.0605 (12)	−0.0022 (8)	0.0281 (9)	0.0077 (9)
C7	0.0373 (8)	0.0359 (8)	0.0357 (8)	0.0060 (6)	0.0140 (6)	0.0007 (7)
C8	0.0372 (8)	0.0404 (9)	0.0374 (9)	−0.0008 (7)	0.0092 (7)	−0.0037 (7)
C9	0.0365 (8)	0.0414 (9)	0.0447 (10)	−0.0024 (7)	0.0138 (7)	0.0015 (7)
C10	0.0489 (10)	0.0457 (10)	0.0380 (9)	0.0053 (8)	0.0139 (7)	0.0037 (7)
C11	0.0478 (10)	0.0528 (11)	0.0490 (11)	−0.0034 (8)	0.0141 (8)	0.0027 (9)
C12	0.0447 (10)	0.0735 (14)	0.0561 (12)	0.0056 (10)	0.0179 (9)	0.0034 (10)
C13	0.0582 (12)	0.0620 (13)	0.0538 (12)	0.0172 (10)	0.0141 (10)	0.0006 (10)
C14	0.0844 (17)	0.0455 (12)	0.114 (2)	0.0059 (11)	0.0467 (16)	0.0118 (13)
C15	0.0691 (14)	0.0508 (12)	0.104 (2)	0.0064 (10)	0.0505 (14)	0.0160 (12)
C16	0.0631 (13)	0.0690 (15)	0.0804 (17)	−0.0278 (12)	0.0166 (12)	−0.0014 (12)
C17	0.0891 (19)	0.0856 (19)	0.091 (2)	0.0387 (16)	0.0270 (16)	−0.0044 (16)
N	0.0482 (8)	0.0456 (8)	0.0419 (8)	0.0030 (7)	0.0191 (7)	0.0048 (6)
O1	0.0558 (8)	0.0645 (9)	0.0548 (9)	−0.0203 (7)	0.0143 (7)	0.0108 (7)
O2	0.0675 (9)	0.0912 (12)	0.0436 (8)	−0.0247 (9)	0.0102 (7)	0.0182 (8)
O3	0.0841 (10)	0.0512 (8)	0.0418 (8)	0.0043 (7)	0.0092 (7)	−0.0097 (6)
O4	0.0925 (12)	0.0847 (11)	0.0534 (9)	0.0253 (10)	0.0450 (9)	0.0168 (8)
S	0.0639 (3)	0.0513 (3)	0.0347 (3)	0.0115 (2)	0.0198 (2)	0.00229 (18)

### Geometric parameters (Å, º)

**Table:**

C1—O2	1.417 (3)	C10—C11	1.382 (3)
C1—O1	1.424 (3)	C10—S	1.7539 (19)
C1—H1B	0.9700	C11—C12	1.379 (3)
C1—H1A	0.9700	C11—H11	0.9300
C2—C3	1.363 (3)	C12—C13	1.379 (3)
C2—O2	1.373 (2)	C12—H12	0.9300
C2—C9	1.392 (3)	C13—C14	1.382 (3)
C3—C4	1.404 (3)	C13—C17	1.503 (3)
C3—H3	0.9300	C14—C15	1.374 (3)
C4—C7	1.406 (2)	C14—H14	0.9300
C4—C5	1.445 (3)	C15—H15	0.9300
C5—C6	1.341 (3)	C16—H16A	0.9600
C5—C16	1.499 (3)	C16—H16B	0.9600
C6—N	1.414 (2)	C16—H16C	0.9600
C6—H6	0.9300	C17—H17A	0.9600
C7—C8	1.398 (2)	C17—H17B	0.9600
C7—N	1.409 (2)	C17—H17C	0.9600
C8—C9	1.366 (2)	N—S	1.6589 (16)
C8—H8	0.9300	O3—S	1.4264 (16)
C9—O1	1.374 (2)	O4—S	1.4245 (16)
C10—C15	1.381 (3)		
			
O2—C1—O1	108.90 (17)	C13—C12—C11	121.5 (2)
O2—C1—H1B	109.9	C13—C12—H12	119.2
O1—C1—H1B	109.9	C11—C12—H12	119.2
O2—C1—H1A	109.9	C12—C13—C14	118.2 (2)
O1—C1—H1A	109.9	C12—C13—C17	121.2 (2)
H1B—C1—H1A	108.3	C14—C13—C17	120.6 (2)
C3—C2—O2	127.80 (17)	C15—C14—C13	121.5 (2)
C3—C2—C9	122.80 (17)	C15—C14—H14	119.2
O2—C2—C9	109.39 (16)	C13—C14—H14	119.2
C2—C3—C4	115.51 (16)	C14—C15—C10	119.2 (2)
C2—C3—H3	122.2	C14—C15—H15	120.4
C4—C3—H3	122.2	C10—C15—H15	120.4
C3—C4—C7	120.70 (16)	C5—C16—H16A	109.5
C3—C4—C5	131.16 (16)	C5—C16—H16B	109.5
C7—C4—C5	108.13 (16)	H16A—C16—H16B	109.5
C6—C5—C4	107.02 (16)	C5—C16—H16C	109.5
C6—C5—C16	127.80 (19)	H16A—C16—H16C	109.5
C4—C5—C16	125.17 (19)	H16B—C16—H16C	109.5
C5—C6—N	110.52 (17)	C13—C17—H17A	109.5
C5—C6—H6	124.7	C13—C17—H17B	109.5
N—C6—H6	124.7	H17A—C17—H17B	109.5
C8—C7—C4	123.30 (16)	C13—C17—H17C	109.5
C8—C7—N	129.75 (15)	H17A—C17—H17C	109.5
C4—C7—N	106.87 (15)	H17B—C17—H17C	109.5
C9—C8—C7	113.86 (15)	C7—N—C6	107.42 (15)
C9—C8—H8	123.1	C7—N—S	126.55 (13)
C7—C8—H8	123.1	C6—N—S	121.92 (13)
C8—C9—O1	126.47 (16)	C9—O1—C1	105.71 (15)
C8—C9—C2	123.82 (16)	C2—O2—C1	106.13 (16)
O1—C9—C2	109.70 (16)	O4—S—O3	120.32 (10)
C15—C10—C11	120.45 (19)	O4—S—N	105.29 (10)
C15—C10—S	119.21 (16)	O3—S—N	106.38 (8)
C11—C10—S	120.33 (15)	O4—S—C10	108.84 (9)
C12—C11—C10	119.1 (2)	O3—S—C10	109.32 (10)
C12—C11—H11	120.5	N—S—C10	105.67 (8)
C10—C11—H11	120.5		
			
O2—C2—C3—C4	−179.98 (19)	C17—C13—C14—C15	−179.5 (3)
C9—C2—C3—C4	0.3 (3)	C13—C14—C15—C10	−0.2 (4)
C2—C3—C4—C7	0.4 (3)	C11—C10—C15—C14	−0.1 (4)
C2—C3—C4—C5	−178.72 (19)	S—C10—C15—C14	−179.0 (2)
C3—C4—C5—C6	178.8 (2)	C8—C7—N—C6	−178.99 (17)
C7—C4—C5—C6	−0.3 (2)	C4—C7—N—C6	−2.23 (18)
C3—C4—C5—C16	0.3 (3)	C8—C7—N—S	23.7 (3)
C7—C4—C5—C16	−178.9 (2)	C4—C7—N—S	−159.50 (13)
C4—C5—C6—N	−1.1 (2)	C5—C6—N—C7	2.1 (2)
C16—C5—C6—N	177.4 (2)	C5—C6—N—S	160.66 (14)
C3—C4—C7—C8	−0.6 (3)	C8—C9—O1—C1	178.0 (2)
C5—C4—C7—C8	178.63 (16)	C2—C9—O1—C1	−1.6 (2)
C3—C4—C7—N	−177.66 (16)	O2—C1—O1—C9	3.5 (3)
C5—C4—C7—N	1.60 (19)	C3—C2—O2—C1	−176.7 (2)
C4—C7—C8—C9	0.2 (2)	C9—C2—O2—C1	3.0 (2)
N—C7—C8—C9	176.48 (16)	O1—C1—O2—C2	−4.0 (3)
C7—C8—C9—O1	−179.05 (17)	C7—N—S—O4	−164.49 (15)
C7—C8—C9—C2	0.5 (3)	C6—N—S—O4	41.24 (17)
C3—C2—C9—C8	−0.8 (3)	C7—N—S—O3	−35.75 (17)
O2—C2—C9—C8	179.44 (18)	C6—N—S—O3	169.99 (15)
C3—C2—C9—O1	178.81 (18)	C7—N—S—C10	80.39 (16)
O2—C2—C9—O1	−0.9 (2)	C6—N—S—C10	−73.87 (16)
C15—C10—C11—C12	0.0 (3)	C15—C10—S—O4	−24.4 (2)
S—C10—C11—C12	178.91 (15)	C11—C10—S—O4	156.74 (16)
C10—C11—C12—C13	0.4 (3)	C15—C10—S—O3	−157.60 (19)
C11—C12—C13—C14	−0.7 (4)	C11—C10—S—O3	23.51 (18)
C11—C12—C13—C17	179.4 (2)	C15—C10—S—N	88.3 (2)
C12—C13—C14—C15	0.6 (4)	C11—C10—S—N	−90.61 (17)

### Hydrogen-bond geometry (Å, º)

Cg1 and Cg2 are the centroids of the (O1,O2,C1,C2,C9) and (C2–C4,C7–C9) rings, respectively.

**Table:**

*D*—H···*A*	*D*—H	H···*A*	*D*···*A*	*D*—H···*A*
C11—H11···*Cg*2^i^	0.93	2.88	3.662 (2)	142
S—O3···*Cg*1^i^	1.42 (1)	3.77 (1)	4.9921 (12)	144 (1)
S—O4···*Cg*1^ii^	1.43 (1)	3.86 (1)	4.9243 (12)	132 (1)

Symmetry codes: (i) −*x*+1, *y*, −*z*+1/2; (ii) *x*+1, −*y*, *z*−1/2.
